# Light-Induced Translocation of RGS9-1 and Gβ5L in Mouse Rod Photoreceptors

**DOI:** 10.1371/journal.pone.0058832

**Published:** 2013-03-28

**Authors:** Mei Tian, Marisa Zallocchi, Weimin Wang, Ching-Kang Chen, Krzysztof Palczewski, Duane Delimont, Dominic Cosgrove, You-Wei Peng

**Affiliations:** 1 Retinal Neurobiology Lab, Boys Town National Research Hospital, Omaha, Nebraska, United States of America; 2 National Institute for Radiological Protection, Chinese Center for Disease Control and Prevention, Beijing, China; 3 Department of Biochemistry & Molecular Biology Virginia Commonwealth University, Richmond, Virginia, United States of America; 4 Department of Pharmacology, School of Medicine, Case Western Reserve University, Cleveland, Ohio, United States of America; University of Oldenburg, Germany

## Abstract

The transducin GTPase-accelerating protein complex, which determines the photoresponse duration of photoreceptors, is composed of RGS9-1, Gβ5L and R9AP. Here we report that RGS9-1 and Gβ5L change their distribution in rods during light/dark adaptation. Upon prolonged dark adaptation, RGS9-1 and Gβ5L are primarily located in rod inner segments. But very dim-light exposure quickly translocates them to the outer segments. In contrast, their anchor protein R9AP remains in the outer segment at all times. In the dark, Gβ5L's interaction with R9AP decreases significantly and RGS9-1 is phosphorylated at S^475^ to a significant degree. Dim light exposure leads to quick de-phosphorylation of RGS9-1. Furthermore, after prolonged dark adaptation, RGS9-1 and transducin Gα are located in different cellular compartments. These results suggest a previously unappreciated mechanism by which prolonged dark adaptation leads to increased light sensitivity in rods by dissociating RGS9-1 from R9AP and redistributing it to rod inner segments.

## Introduction

The duration of a photoresponse determines the sensitivity and speed of vision. It starts when light activates rhodopsin. Activated rhodopsin catalyzes GDP/GTP exchange on transducin α (Tα). The GTP-bound Tα then activates the cGMP-specific phosphodiesterase resulting in cGMP hydrolysis. The decrease in cGMP concentration closes cGMP-gated channels, leading to membrane hyperpolarization [1]–[6]. Multiple steps occur concomitantly during the recovery phase of phototransduction, the slowest of these reactions determines the rate of photoresponse recovery. This rate-limiting step is GTP hydrolysis by Tα [7], which is catalyzed by a GTPase-accelerating protein (GAP) complex consisting of RGS9-1 [8], [9], Gβ5L [10], [11] and R9AP [12], [13].

In this GAP complex, RGS9-1 accelerates GTP hydrolysis by Tα [8]. The association of RGS9-1 with Gβ5L confers mutual stability [14] and strengthens the GAP activity of RGS9-1 [15], [16]. RGS9-1 and Gβ5L depend on the interaction through the DEP domain of RGS9-1 with a membrane protein R9AP to anchor them to the disk membrane [12], [17]. The binding of RGS9-1 to R9AP requires Gβ5L [18]. Furthermore, R9AP not only anchors the RGS9-1-Gβ5L complex to membranes, it also enhances the ability of RGS9-1 to stimulate the GTPase activity of Tα [12], [19], [20]. Therefore, three members of the GAP complex must work together to ensure efficient transducin turn-off. When they dissociate, RGS9-1′s activity on Tα could be significantly reduced and the duration of photoresponses extended.

It has been reported that RGS9-1 is robustly phosphorylated in the dark, light exposure dampens this phosphorylation. In mouse rods RGS9-1 is phosphrylated at Ser^475^ by PKCα. *In vitro,* phosphorylated RGS9-1 has a decreased affinity for R9AP [21]–[23]. Thus, it appears that dark adaptation promotes phosphorylation of RGS9-1 and dissociates it from R9AP.

Here, we report for the first time that, RGS9-1 and Gβ5L change their location in rods during light/dark adaptation. Upon prolonged dark adaptation, RGS9-1 and Gβ5L are both situated in the inner segments. Light activates their redistribution to the outer segments. In contrast, R9AP remains located in the outer segments regardless of lighting conditions. These results suggest that upon prolonged dark adaptation, RGS9-1 and R9AP may be separated in the rods. Consistently, we found that, after dark adaptation, the interaction between R9AP and Gβ5L was significantly weakened, and RGS9-1 is phosphorylated. Very dim light exposure led to pronounced RGS9-1 dephosphorylation. These results demonstrate that there is a mechanism in rods to separate RGS9-1 from transducin during dark adaptation. This mechanism may be used to increase sensitivity of photoreceptor at the expense of reduced temporal resolution.

## Methods

### Ethics Statement

All animal handling and procedures were performed in accordance with protocols for these studies that have been approved by the Boys Town National Research Hospital Institutional Animal Care and Use Committee (IACUC).

### Animals

Wild type pigmented 129 Sv/J mice of either sex were used for all studies. The retinoid isomerase RPE65 transcript for this strain was amplified and sequenced and found to be of the L450 genotype for RPE65. The animals were kept at the Boys Town National Research Hospital (BTNRH) *vivarium* in transparent cages under 12 hr. light (about 200 lux)/dark cycle. Procedures for handling animals followed NIH guidelines and were in accordance with an approved institutional BTNRH IACUC protocol. Every effort was made to minimize their discomfort and distress. Procedures for light/dark adaptation did not cause pain, discomfort, distress or morbidity. The animals were anesthetized with a mixture of ketamine 300 mg/kg and xylazine 30 mg/kg body weight, administered IP, prior to euthanizing by cervical dislocation to eliminate the potential for pain. Tissues were obtained after the animals were euthanized.

### Antibodies

Antibodies against the following proteins were used: rhodopsin and β-actin (Sigma, MO), the α subunit of rod transducin (CytoSignal, CA) and synaptophysin (Sigma, MO). Anti-Phospho-RGS9 antibodies were generated as described previously [23]. Three anti-RGS9-1 antibodies against different epitopes of RGS9-1 were used. The first (CT318, against the C-terminal of RGS9-1) was produced by Dr. C-K. J. Chen [9]; the second (against residue 226–484 of RGS9-1) was generated by Dr. T. Wenzel, Baylor College of Medicine [8], and the third was chicken anti-RGS9-1 (Sigma, against residue 20–200 of RGS9). Two antibodies against different epitopes of Gβ5L were used. The first specifically recognizing only the long form of Gβ5 (sc-14947, against a region close to the N-terminal of Gβ5L) is from Santa Cruz Biotechnology (Santa Cruz, CA). The second (CT215, against the very end of N-terminal of Gβ5 short form) that recognizes both the short and long splice forms was a gift from Dr. Mel Simon of Caltech [24]. Anti-R9AP antibody was a gift from Dr. V. Arshavsky, Duke University.

### Light/Dark Adaptation

For dark adaptation, the animals were kept in cages without any restraint in a lightproof darkroom devoid of detectable light. Because very dim light exposure (less than 1 lux) can activate the translocation of RGS9-1 and Gβ5L to rod outer segments, we, developed a specialized dark adaptation environment to prevent this activation. The animals were kept in cages in this lightproof darkroom of complete darkness without any detectable light. The detectable light includes: 1. Electronic signal lights from any machine. 2. Any detectable light from the ceiling or doors after sitting in the darkroom for more than 30 min. 3. The light reflecting from the eye contacts of infrared cameras. After dark adaptation, the retinas were isolated and processed either under complete darkness or under very dim light depending on the experiment. For experiments under complete darkness, the retina must be isolated and processed in complete darkness, including animal euthanasia, isolating the retina and photoreceptors, and processing the retina. The most important point is that if the photoreceptor is exposed to even very dim light during the isolation process, RGS9-1 and Gβ5L will be quickly translocated back to the outer segment and it will take another 8-hours dark adaptation to translocate it down to the inner segment. The experimental manipulations were done in complete darkness or under very dim light as required for each individual experiment. For light adaptation, animals were first kept in the darkroom for 8 hours, and then exposed to various light intensities in transparent cages. Light intensities were measured inside the cages. The light sources (diffuse white fluorescent light) were placed 4–6 inches above the cages and beside the cages on all four sides.

### Immunocytochemistry

This methodology has been described in detail in previous publications [25]–[27]. Briefly, eyes were quickly removed from mice euthanized under deep anesthesia with 2,2,2-tribromoethanol (Avertin, Sigma MO) at a dose of 300 µg/g body weight. After removal of the anterior segments, posterior eyecups were fixed in 4% paraformaldehyde in 100 mM sodium phosphate buffer (PB, pH 7.3) at 4°C. The concentration of paraformaldehyde and time of fixation was varied according to the immunoreactivity of various antigens. The tissue was transferred sequentially into 5% and 30% sucrose in PB, each at 4°C overnight. Retinal sections (2–8 μm thickness), were cut with a Microm cryostat and mounted on gelatin-coated slides. Retinal sections were first incubated with 5% normal goat serum (Vector Laboratories) in PBS for 1 hour at room temperature, and then incubated with primary antibodies overnight at 4°C [anti-transducin α1 antibody (Cytosignal, CA) 1∶1000; anti-RGS9 1∶1000; anti-R9AP 1∶1000; anti-rhodopsin 1∶1000], followed by three washes in PBS of 15 min. each. The sections were then incubated with either Alexa 594-conjugated anti-mouse immunoglobulin antibody (Invitrogen, Eugene, Eugene, OR) 1∶250, Alexa 488-conjugated anti-rabbit immunoglobulin antibody (Invitrogen, Eugene, OR) 1∶250 or Alexa 594-conjugated anti-goat immunoglobulin antibody (Invitrogen, Eugene, OR) 1∶250 for 2 hours at room temperature. Resulting slides were washed with PBS and coverslipped with 50% glycerol in PBS for viewing using a Zeiss AX10 fluorescent microscope. For double-immunostaining, retinal sections were incubated with mixed primary and secondary antibodies. All incubation and wash buffers contained 0.3% Triton X-100.

### Serial tangential sectioning and immunoblotting

The method was modified based on Sokolov and Martemyanov [17], [28]. After dark adaptation, mouse retinas isolated under either light or dim light were suspended in ice-cold Ringer's solution and immediately flat-mounted on PVDF membranes, placed between two glass slides separated by 0.5 mm spacers and immediately frozen (as fast as possible). For sectioning, the glass slides were separated, and the PVDF membrane attached retina was mounted on a cryomicrotome specimen holder. Sequential 5 µm tangential retinal sections were collected in 100 µl of SDS-PAGE sample buffer. For dim light experiments, all the procedures before putting in sample buffer were performed under very dim light (20 or <20 lux). Aliquots of each sample were subjected to 12% of SDS-PAGE followed by Western Blotting (see below). The procedures for serial tangential section immunoblotting were highly standardized. We ensured that all retina samples (a round patch after trimming all the curved edges from a whole mouse retina) had a similar size (around 2 mm); the proper mounting and orientation of the retina was controlled by the location of the standard protein markers rhodopsin and synaptophysin. When properly mounted, rhodopsin is present only in the first 1–7 sections and synaptophysin is present in sections of much higher number far away from rhodopsin (if the retina is not properly oriented, rhodopsin will appear in other sections in the regions of inner segments, INL or OPL and synatophysin will also appear in the earlier sections); rhodopsin must be at least in the first 5–6 sections to ensure the appropriate thickness of photoreceptor outer segment layer; each section is of the same thickness and collected in the same amount of sample buffer; the protein concentration of each retina sample in different experiments was measured to ensure they are similar; for each lighting condition, at least 4 replicate experiments were performed that met with the above requirements.

### Phosphorylation of RGS9 on Ser^475^ immunoblot

Mouse retinas were isolated and homogenized in 50 µl of RIPA buffer containing 2 mM NaF, 1 mM Na_3_VO_4_, protease inhibitors (Sigma MO, P8340) and 1 µM microcystin-LR (a specific inhibitor of protein phosphatase 1 (PP1) and PP2A). Homogenates were cleared by centrifugation at 10,000 rpm for 10 min at 4°C and 15 µg of protein (Bio-Rad CA, 500–0006) were resolved on 10% SDS-PAGE gels under reducing conditions, transferred to PVDF membranes for 1 h at 100 V and blocked overnight at 4°C with rocking. For total RGS9 (Sigma MO, GW22901) and β-actin (Sigma MO, A5441) the blocking solution was 10% milk (10% glycerol, 9% NaCl, 10% non-fat milk, 0.2% Tween-20 in PBS) and the antibodies were diluted 1/1,000 in the same blocking solution and incubated overnight at 4°C with rocking. After two washes total RGS9 immunoblotted membranes were incubated with anti-chicken biotinylated antibodies 1/500 (Vector Lab. CA, BA9010) followed by Streptavidin-HRP (Bio-Rad CA, 80709) 1/500, in 10% milk blocking solution, each for 1 hour at room temperature. The β-actin immunoblotted membranes were incubated with anti-mouse HRP conjugated antibody (Sigma MO, A5278) 1/2,000 dilution. For phospho-RGS9 (ascites called D3), membranes were blocked with 5% BSA blocking solution (100 mM NaCl, 0.2% Tween-20, 5% BSA in 10 mM Tris pH 7.4) and the antibody was diluted 1/50 in the same blocking solution and incubated overnight at 4°C with rocking. After two washes, the membranes were incubated with Protein G-HRP conjugated (Millipore MA, 18–161) 1/20,000 in 5% BSA for 1 h at room temperature. After four washes, specific protein bands were detected with the ECL system (Pierce IL, 32106).

### Immunoprecipitation

Whole retinas were removed from mice and homogenized in IP lysis buffer containing 25 mM Tris•HCl pH 7.4, 150 mM NaCl, 1%NP-40, 1 Mm EDTA, 5% glycerol and protease inhibitor cocktail (Sigma,P8340). Homogenates were cleared by centrifugation at 12,000 rpm for 10 min at 4°C and 150 μg of protein were pre-cleared by 15 μl TrueBlot anti-goat Ig IP beads (eBioscience, 00–8844) on ice for 30 min, and then spun at 10,000 g. The supernatant was collected and 1 μg anti-mouse Gβ5L antibody (Santa Cruz, sc-14947) was added to pre-clear lysate and incubated at 4°C overnight on a rotator, followed by addition of 20 μl anti-goat Ig IP beads and incubate for 3 hours. After centrifuging for 10 min at 4°C, the supernatant was carefully removed and the beads were washed 3 times with 500 μl IP lysis buffer. Protein bound to the beads was eluted with an SDS page loading buffer (62 mM Tris, 10% glycerol, 2% SDS, and 2%β-mercaptoethanol) and boiled for 10 min. The supernatant was collected and loaded onto PAGE gels. In control experiments, immunoprecipitation was performed similarly either by using IP lysis buffer instead of retina lysate or adding normal goat IgG to retina lysate as the IP antibody (instead of goat anti-Gβ5L antibody). Protein bands were transferred onto PVDF membranes and analyzed by immunoblotting with the following antibodies: rabbit antibody against RGS9-1(CT318), rabbit antibody against R9AP144–223fragment or anti-Gβ5L (A-16) antibody (Santa Cruz). Clean-Blot IP detection reagent (HRP,Thermo Science) was used to detect rabbit IgG and HRP-conjugated anti-goat secondary antibody was used for goat IgG detection.

### Statistical analysis

Semi-quantitative analysis for immunocytochemical staining and western blotting was carried out densitometrically using Image J software (NIH). Mean density and integrated density were used to represent protein expression level in immunocytochemical staining and western blotting respectively, and the data analyzed using the Students t-test.

## Results

### Under light and dark adaptation, RGS9-1 and Gβ5L show a different distribution in mouse rod photoreceptors. In contrast, R9AP is consistently localized in the rod outer segments

**Figure 1:**
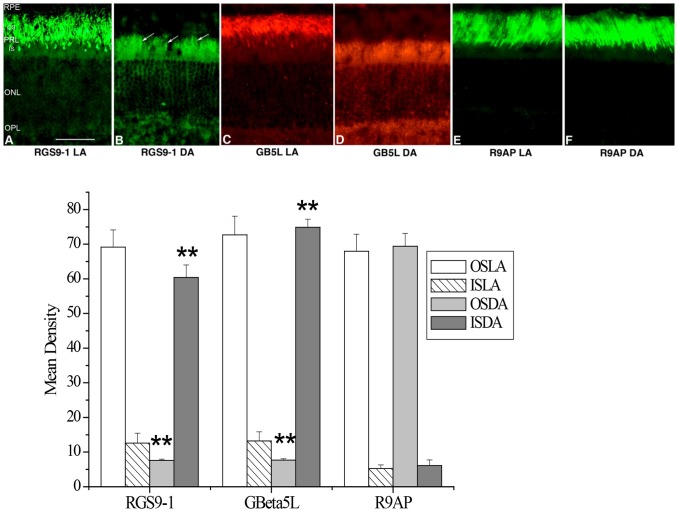
RGS9 and Gβ5L show different distribution in mouse rods in light (LA) and dark adaptation (DA). Upper panel: Immunostaining of RGS9 (A and B), Gβ5L (C and D) and R9AP (E and F) in 200 lux light adapted (A, C and E) and dark adapted (B, D and F) mouse retinas. Arrows indicate labeling of RGS9 in the rod inner segments. RPE = Retinal Epithelium Layer; PRL = Photoreceptor Layer; OS = outer segment; IS = Inner Segment; ONL = Outer Nuclear Layer; OPL = Outer Plexiform Layer; bar = 25 µm. Lower panel: Analysis of the average levels of RGS9-1, Gβ5L and R9AP in the outer segment layer (OS) and inner segment layer (IS). OSLA = outer segment in light adapted condition; ISLA = inner segment in light adapted condition; OSDA = outer segment in dark adapted condition; ISDA = inner segment in dark adapted condition; n = 8; **P<0.001 dark adaptation vs light adaptation.

**Figure 2:**
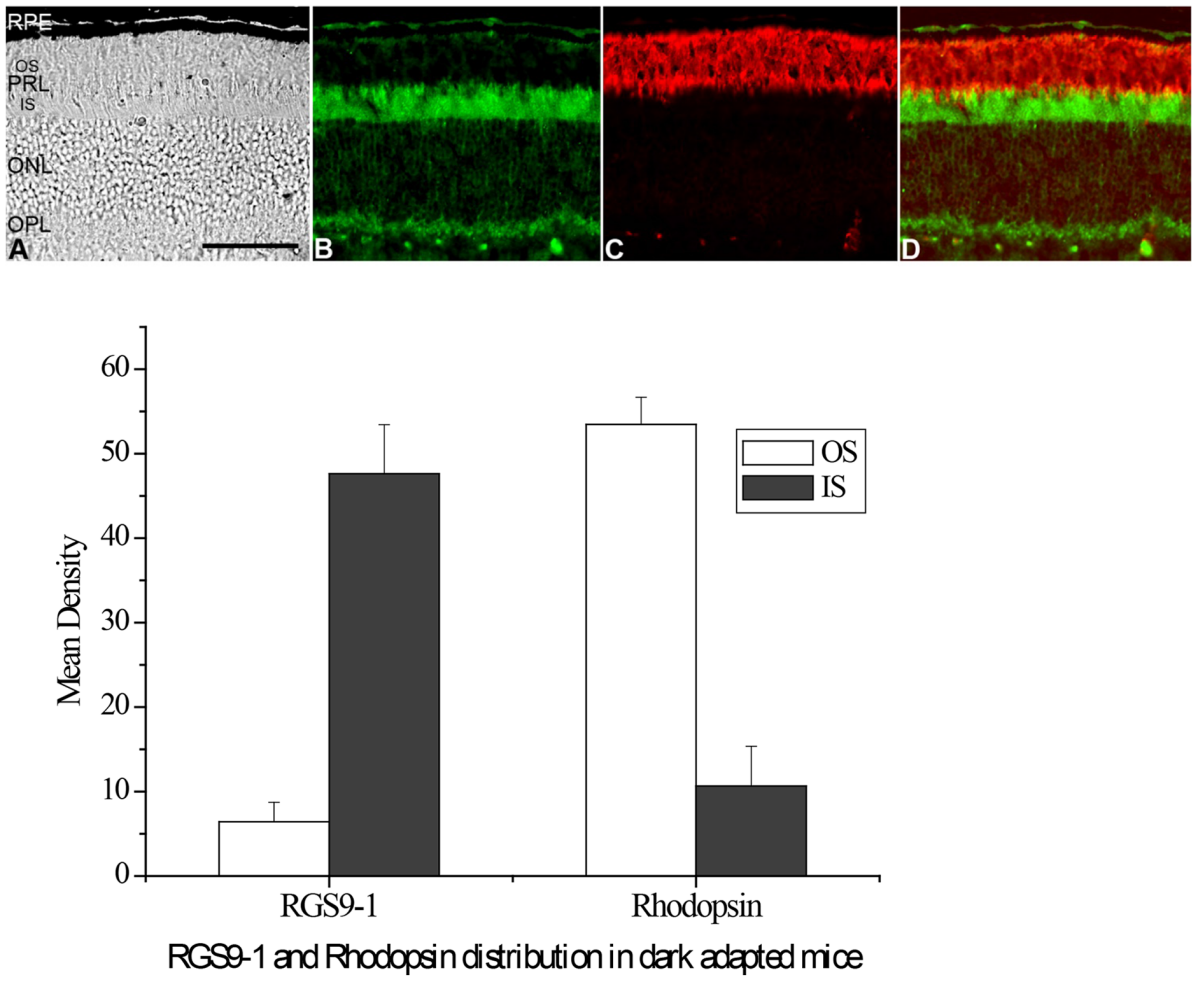
Double labeling studies using anti-RGS9 and anti-rhodopsin antibodies on dark adapted mouse retina isolated and processed in complete darkness. Upper panel: **A**: the DIC image of an 8-hours dark adapted mouse retinal section indicates structural integrity of rod outer segments. This retinal section was used for a double-immunostaining of RGS9 and rhodopsin (can be recognized by the shape of RPE in A, B and D). After prolonged dark adaptation, RGS9-1 (**B**) is located primarily in rod inner segments and rhodopsin (**C**), which, in normal wild type mice, is always located in rod outer segments and is not translocated by light, is located in the rod outer segments. **D**: Double-immunostaining (Merge) of RGS9-1 and rhodopsin in the same region. Lower panel: Analysis of the average levels of RGS9-1 and rhodopsin in the outer segment layer and inner segment layer; n = 8. Labels are the same as in Fig. 1.

**Figure 3:**
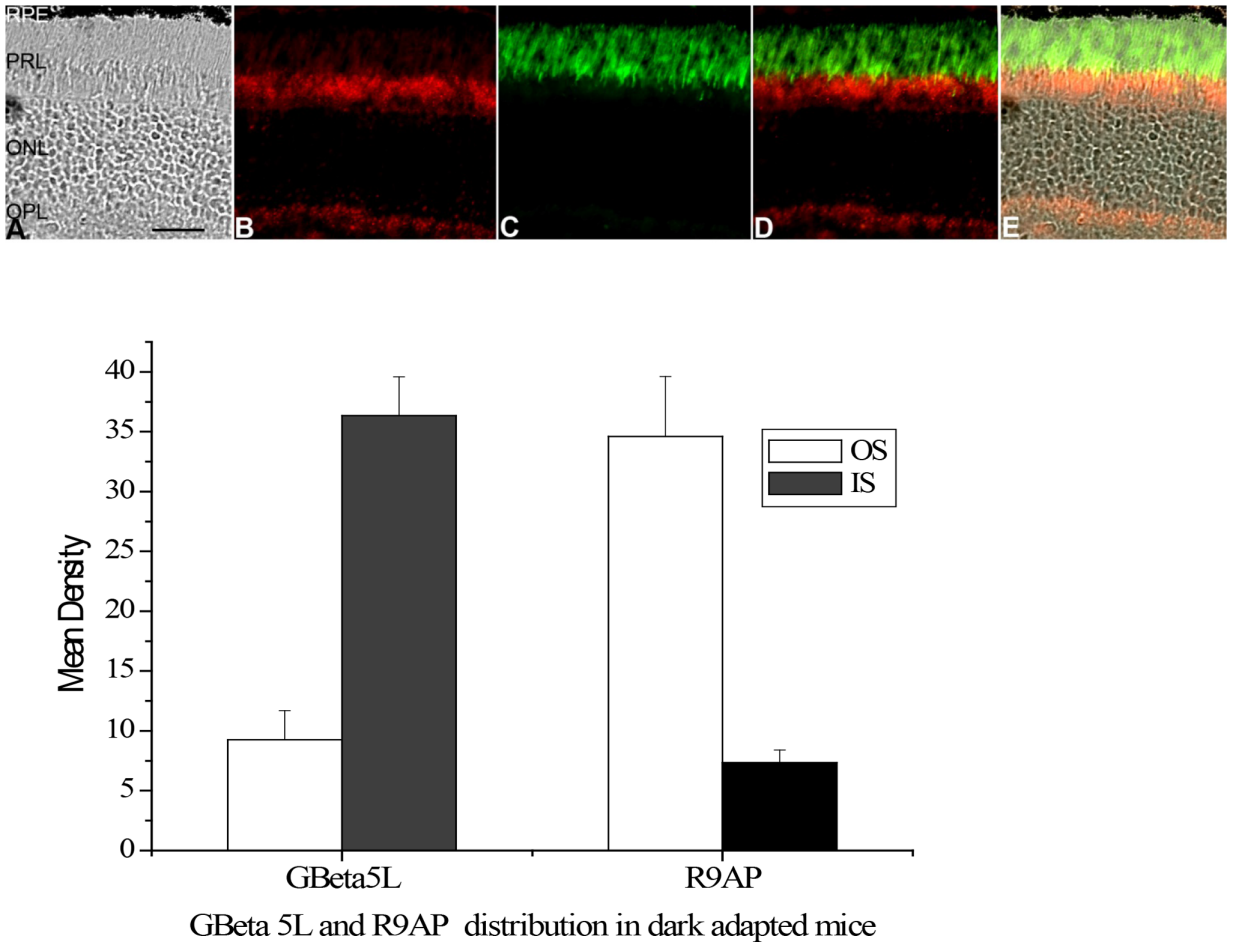
Double labeling studies using anti-Gβ5L and anti-R9AP antibodies on a dark adapted mouse retina isolated and processed in complete darkness. Upper panel: **A**: the DIC image of an 8-hours dark adapted mouse retinal section indicates structural integrity of rod outer segments. This retinal section was used for a double-immunostaining of Gβ5L and R9AP. This figure clearly shows that, after prolong dark adaptation; Gβ5L is located primary in rod inner segments (B) while R9AP is always located in rod outer segments (C). **D**: Double-immunostaining (Merge) of Gβ5L and R9AP in the same region. **E**: Merge of Gβ5L and R9AP under DIC image. Lower panel: Analysis of the average levels of Gβ5L and R9AP in the outer segment layer (OS) and inner segment layer (IS); n = 8. Labels are the same as in Fig. 1.

**Figure 4:**
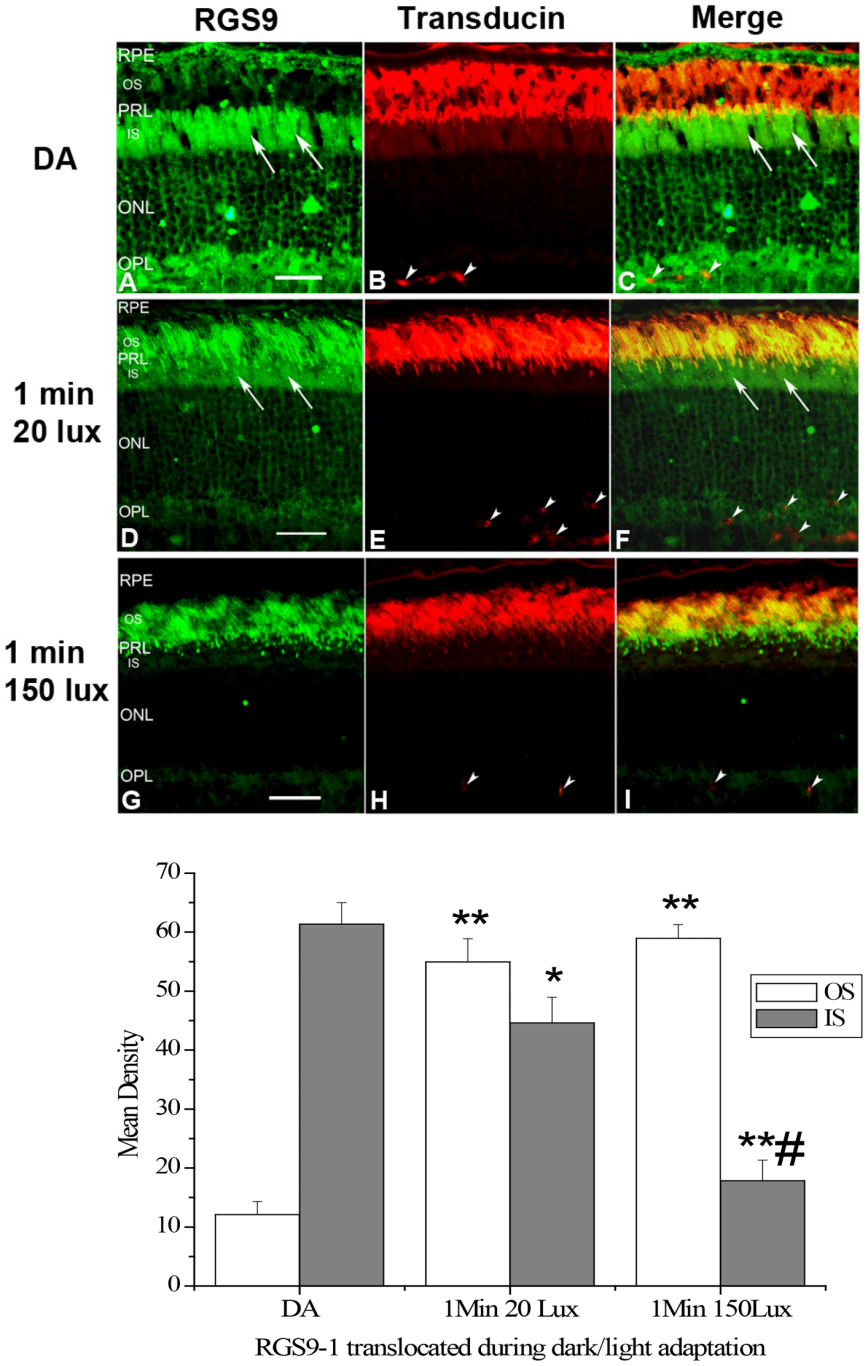
Double labeling studies using anti-RGS9 and anti-Transducin α antibodies confirm that RGS9 is translocated between rod outer segments and inner segments during light/dark adaptation. Upper panel: **A**–**C**: Double immunostaining (Merge in C) of RGS9 (A) and Transducin α (B) on a wild type mouse retina after dark adaptation (DA); **D**–**F**: Double immunostaining (Merge in F) of RGS9 (D) and Transducin α (E) on a wild type mouse retina after 1 min 20 lux light exposure; **G**–**I**: Double immunostaining (Merge in I) of RGS9 (G) and Transducin α (H) on a wild type mouse retina after 1 min 150 lux light exposure. Arrows indicate labeling of RGS9 in the inner segments. Arrow heads indicate non-specifically labeled blood vessels. Other labels are the same as in Fig. 1. Lower panel: Analysis of the average levels of RGS9-1 in the outer segment layer (OS) and inner segment layer (IS); n = 8. *P<0.01,**P<0.001 vs DA; #P<0.001 vs 1 Min 20Lux.

Formerly, all three members in the transducin GAP complex were considered an outer segment resident protein, as shown in Figs. 1A, 1C and 1E when the retina was light adapted. However, we found that, after a prolonged dark adaptation, RGS9-1 and Gβ5L was located mostly in the inner segment layer when the retinas were isolated and processed under complete darkness (Figs. 1B and 1D). In contrast, the membrane anchor protein of the GAP complex, R9AP, remained always localized to the outer segments regardless of lighting conditions (Figs. 1E and 1F). Comparing to R9AP, the levels of RGS9-1 and Gβ5L in outer segment layer and inner segment layer in dark adapted and light adapted conditions clearly showed significant changes in immunostaining intensity (Fig. 1 lower panel). The change in RGS9-1 and Gβ5L distribution was not an artifact because same results were obtained using additional antibodies against different epitopes of RGS9-1 and Gβ5L (data not shown). To test whether lost or damaged outer segment layer in sections contributed to this finding, we performed double-labeling experiments with 1) anti-rhodopsin and anti-RGS9-1 antibodies (Fig. 2); 2) anti- Gβ5L and anti-R9AP antibodies (Fig. 3); and 3) anti-RGS9-1 and anti-transducin antibodies (Fig. 4A–C). The relative levels of RGS9-1, Gβ5L and R9AP in outer segment and inner segment layers in these double-labeling experiments were semi-quantitatively analyzed. The results confirmed that RGS9-1 and Gβ5L was indeed differentially distributed in rods after light and dark adaptations. This proves that the lack of RGS9-1 staining in the outer segment layer is not due to absence of outer segments but indeed results from light-dependent redistribution of RGS9-1 and Gβ5L in rods. Most strikingly, after dark adapting the animals for 8 hours, RGS9-1 and transducin were located in different cellular compartments (Fig. 4A–C).

### The threshold for RGS9-1′s translocation back into the outer segment is much lower than that required for transducin′s translocation to the inner segment

We further found that a 1-min exposure to 20 lux light was sufficient to trigger a significant RGS9-1 translocation back to outer segments. Importantly, double labeling studies (Figs. 4D–F) revealed that a significant amount of RGS9-1 still remained in the inner segment compartment. This indicated that under such dim light conditions RGS9-1 was located in both outer and inner segments. This observation also suggested that, under such low light intensity, RGS9-1 was moving from the inner segments to the outer segments, and further confirmed that RGS9's distribution in rods can be changed in response to light/dark adaptation. Interestingly, Fig. 4D–F indicated that 1 min of 20 lux light activated RGS9-1's translocation but not that of transducin. To explore whether the threshold for RGS9-1 translocation of is indeed lower than that for transducin, we elevated the light intensity to 150 lux. After a 1-min exposure we found that most of the RGS9-1 had already clearly localized in the rod outer segment compartment (Fig 4G). At the same time, double labeling of the same retinal section with anti-transducin α and anti-RGS9 antibodies revealed that, after 1 min of 150 lux light exposure, transducin α was still mostly located in the rod outer segments (Figure 4H). These results suggest that the light activation threshold for RGS9 translocation is lower than that for transducin. Differences in threshold for translocation of transducin and its GAP complex may conceivably have important functions, which will be speculated in the Discussion.

### Very dim light exposure quickly activates the translocation of RGS9/Gβ5L back to rod outer segments

**Figure 5:**
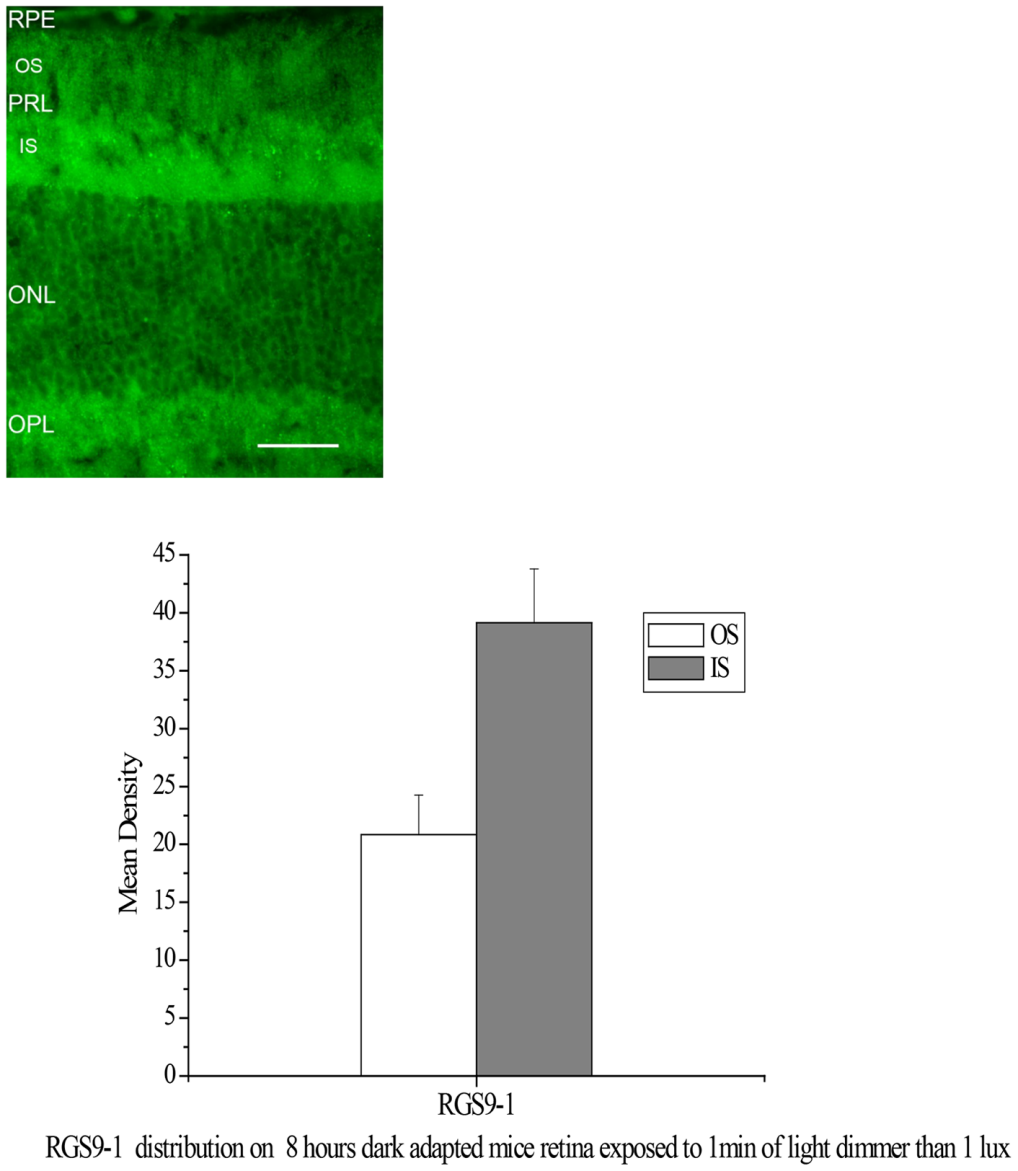
Immunostaining of RGS9-1 on an 8 hours dark adapted mouse retina exposed to 1 min of light dimmer than 1 lux. Upper panel: There is clear RGS9-1 staining in the rod outer segment layer, though staining in inner segment layer is much stronger. Labels are the same as in Fig. 1. Lower panel: Analysis of the average levels of RGS9-1 in the outer segment layer (OS) and inner segment layer (IS); n = 8. Labels are the same as in Fig. 1.

We found that a minimum of 8 hours of dark adaptation was required for RGS9-1/Gβ5L to localize predominately in the inner segments. However, translocation of RGS9-1/Gβ5L back to the outer segments was rapid, and extremely sensitive to light stimulation. Even in a completely dark room, a 1-min exposure to a light less than 1 lux is sufficient to trigger translocation (Fig. 5). A very dim light, for example, leaking from the ceiling can activate translocation back to the outer segments. This level of light can only be detected after sitting in the darkroom for more than 30 min, and its intensity cannot be detected by the light meter. Thus far, the only method to observe a clearly dominant distribution of RGS9-1 in rod inner segments is immunocytochemistry since this method allowed isolation and processing of retinas in complete darkness.

### Tangential serial section immunoblotting studies confirm that RGS9-1 and Gβ5L are moving from outer segments to inner segments after dark adaptation

**Figure 6:**
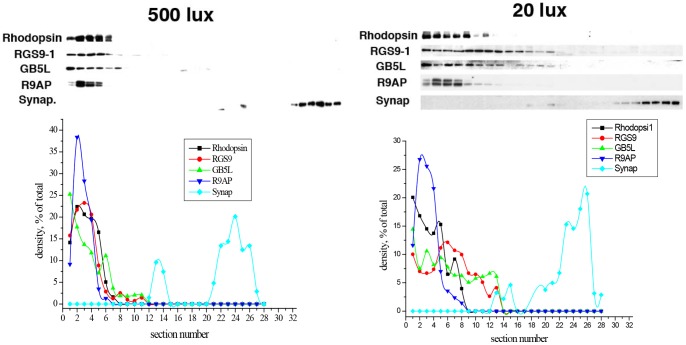
Tangential Serial Section Immunoblotting quantitative analysis of RS9-1, Gβ5L, R9AP in mouse retina. Upper left: Western blots of tangential serial sections of a mouse retina after dark adaptation for 8 hours followed by 500 lux light exposure for 10 min, and then processing under 500 lux light exposure. Upper right: Western blots of tangential serial sections of a mouse retina after being dark adapted for 8 hours and then isolated and processed under dim light exposure (20 lux). The sections were cut starting from the top of the outer segments, and were immunolabeled by specific antibodies against rhodopsin, RGS9-1, Gβ5L, R9AP, and synaptophysin. Rhodopsin and synaptophysin were used as markers for the locations of the outer segments and synaptic regions of the rod photoreceptors. Lower panels: Densitometric profiles of the Western blots from the upper panels in which the densities of individual bands for RGS9, Gβ5L, R9AP, rhodopsin, and synaptophysin were expressed as percentages of the total density of all bands representing each individual protein on the blot.

Given the novelty of this observation, we sought to verify the translocation of the GAP complex in light- and dark-adapted retinas by a more stringent method: tangential serial sectioning followed by immunoblotting [28]. As shown in Figure 6 upper left, after dark-adapted retina was exposed to light (500 lux) for 10 min, the majority (>90%) of RGS9-1, Gβ5L, and R9AP coincide with the outer segment marker rhodopsin (present in the first 5–6 sections) and completely separated from synaptophysin signal used here as a basic marker for photoreceptor synapses. After complete dark adaptation of mice for 8 hours and processing and sectioning the retinas under very dim light (20 lux, it is very difficult to do tangential sectioning under light conditions lower than 20 lux), RGS9-1/Gβ5L showed a drastically different distribution. About 50% of the two proteins had moved into the inner segment layer, while R9AP remained similarly localized in the outer segments (first 5–6 sections) as that in light adapted condition (Fig. 6 upper right). These data are also consistent with aforementioned immunocytochemical results (Fig. 4D) showing that when the retina was isolated and processed under 20 lux light exposure, RGS9-1 was distributed in both rod outer and inner segments. These results give further credence to the claim that light-dependent RGS9-1/Gβ5L translocation indeed occurs and that this phenomenon is not due to epitope masking or other unknown artifacts.

### R9AP′s interaction with both RGS9-1 and Gβ5L is significantly reduced as the intensity of light exposure decreases

**Figure 7:**
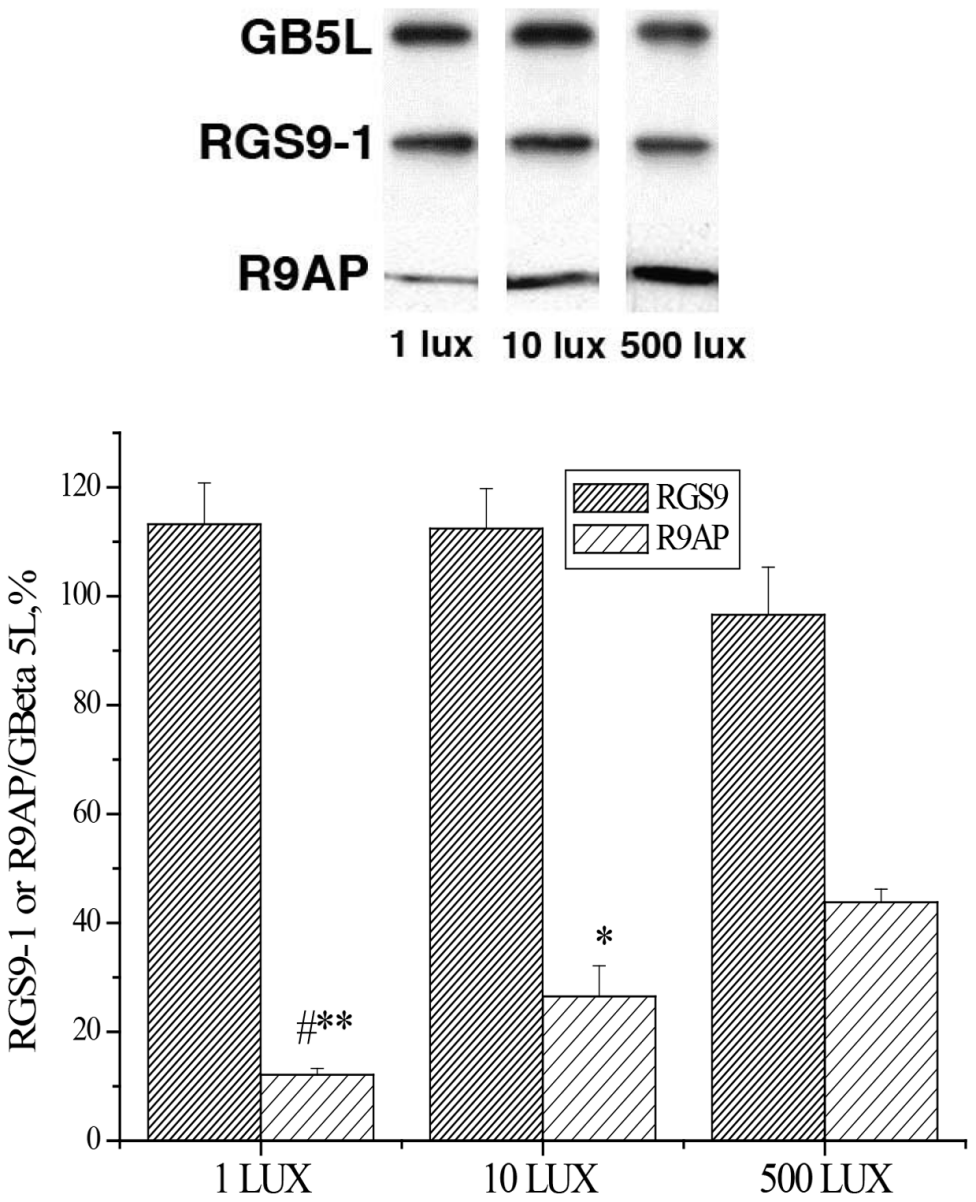
Immunoprecipitation studies indicate that Gβ5L and RGS9-1 are still bound together during light/dark adaptation, and the interaction between the Gβ5L-RGS9-1 complex and R9AP is significantly reduced as the intensity of light exposure is decreased. Mouse retinas were homogenized and subjected to immunoprecipitation (IP) with an anti-Gβ5L antibody (Santa Cruz, CA). The presence of RGS9-1 and R9AP in the eluates after IP was determined by Western blotting with anti-RGS9-1 and anti-R9AP antibodies. Binding levels (lower panel) were measured by using RGS9-1 or R9AP density vs GBeta 5L density. Error bars represent SEM, n = 5. **and * indicate significantly different than 500 lux group at P<0.001 and P<0.05 levels respectively. # indicates significantly different than 10 lux group at P<0.05 level.

RGS9-1 and Gβ5L are obligate partners [9], [14]. Moreover, Gβ5L determines the interaction of RGS9 with the membrane anchor protein R9AP [18]. RGS9-1 binds to Gβ5L directly and this binding does not require R9AP *in vitro* [29], [30]. But our results (Fig. 1–3) suggest that, *in vivo,* RGS9-/Gβ5L in dark-adapted retinas may not be bound to membrane anchor R9AP. We utilized a co-immunoprecipitation approach to further analyze this. As shown in Fig. 7, the amount of R9AP co-precipitated with Gβ5L decreased as the intensities of light exposure was reduced whereas that of RGS9-1 appeared unchanged. Densitometric scanning of the related western blots revealed a ∼4fold drop in the amount of R9AP that co-precipitated with Gβ5L after dark adapted retinas were exposed to 1 vs. 500 lux light intensity. These findings indicate that Gβ5L and RGS9-1 are still bound together during light/dark adaptation, but the interaction between the Gβ5L/RGS9-1 dimer with the membrane anchor R9AP is reduced during dark adaptation, consistent with results obtained by immunocytochemistry and serial tangential sectioning (Figs 1, 2, 3 and 6).

### RGS9-1 is heavily phosphorylated on Ser^475^ upon dark adaptation

**Figure 8:**
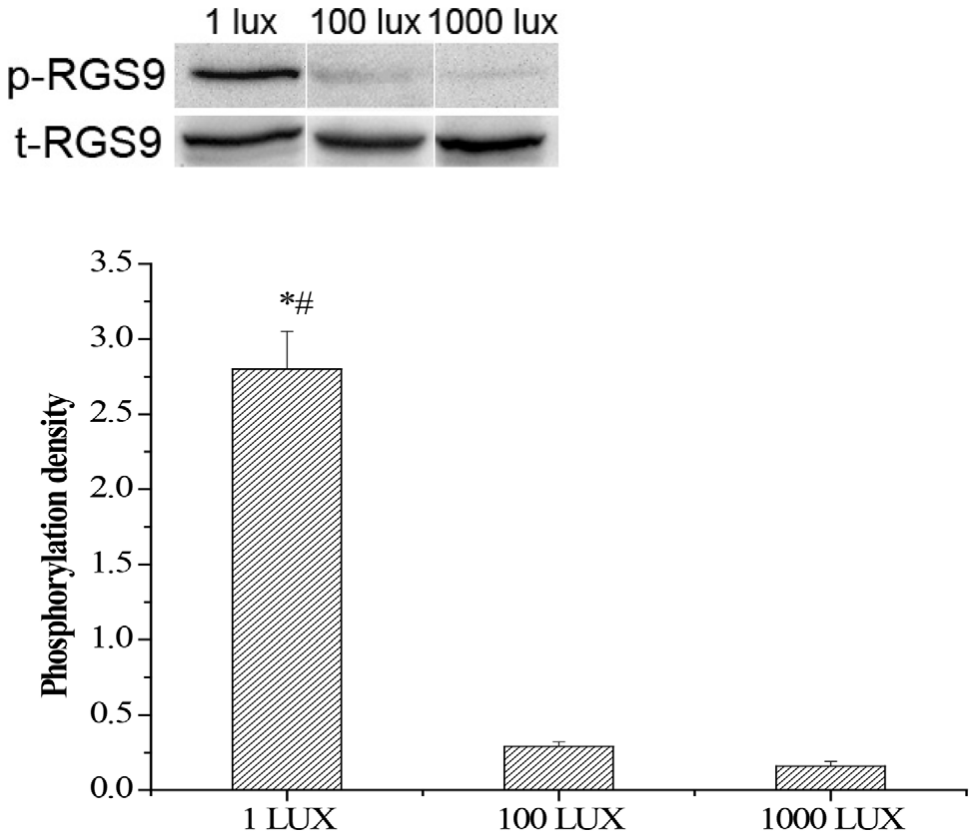
RGS9-1 is heavily phosphrylated on Ser^475^ under very dim light exposure. Upper panel: Mouse retinas isolated under 1 lux, 100 lux and 1000 lux were analyzed by Western blotting probed with a anti-Ser^475^ phospho-RGS9-1 antibody (p-RGS9) and a anti-RGS9 antibody (t-RGS9). The total RGS9 (t-RGS9) is consistent under various light/dark conditions. Lower panel: Densitometric profiles of the densities of the Western blots of individual p-RGS9 bands. Densitometric scans of the autoradiographs were quantified and analyzed statistically. Error bars represent SEM, n = 5. Asterisk denotes statistically significant differences (p<0.05); # indicates significantly different than 1000 lux group at P<0.01 level.

It has been reported that, in mouse rod photoreceptors, RGS9-1 is phosporylated at Ser^475^ by PKC, and such phosphorylation significantly reduces its affinity to R9AP [22], [23]. More importantly, light exposure reportedly reduces the level of phosphorylation of RGS9-1 [21]–[23]. Because we had determined the lighting conditions needed for RGS9-1/Gβ5L translocation in mouse retina, we examined the phosphorylation of RGS9-1 after dark adaptation. With an antibody specific for RGS9-1 Ser^475^ phosphorylation [23], we found that, RGS9-1 was heavily phosphorylated in 8-hour dark adapted mouse retinas, when the retinas were isolated and pro­cessed under very dim light (1 lux). Its level of phosphor­ylation was more than 10 times greater than that observed for RGS9-1 from retinas that had been light (1000 lux) adapted for 10 min and then isolated and processed under 1000 lux illumination conditions. Furthermore, after 10 min 100 lux light exposure, RGS9-1 was quickly dephosphorylated to levels close to that observed for 1000 lux lighting conditions (Figure 8). These results are consistent with the results of immunocytochemistry showing that dim light exposure quickly activates the translocation of RGS9-1 back to rod outer segments. The total levels of retinal RGS9-1 and Gβ5L remained very similar in all lighting conditions, indicating that the changes in phosphorylation and location of RGS9-1 in dark-adapted retina were not due to a change in their expression levels (Fig 8).

## Discussion

### The separability of GAP complex and its target transducin *in vivo* during dark adaptation

Until now, the transducin GAP complex had been viewed as a tight ternary protein complex anchored in the rod outer segment membranes. Several observations helped to establish and sustain this view. First, the components of the GAP complex are obligate partners in that they not only rely on each other for full biological activity, but their interaction also confers mutual intracellular stability. Second, the degree of defects in recovery of rod phototransduction after light exposure is almost identical in RGS9−/−, Gβ5−/−, and R9AP−/− mice, indicating that they all participate in the same reaction during rod recovery. Third, in most immunohistochemical studies published so far, RGS9-1 is largely found in the outer segment compartment of rods, even though it has been shown to be present in other cellular compartments of cones [31]. Here, we investigated whether the localization of transducin GAP complex is dependent on lighting conditions and found that, after prolonged dark adaptation, the majority of RGS9-1/Gb5L complex is located separately from R9AP and transducin. This hitherto unknown cellular localization of RGS9-1/Gβ5L is highly sensitive to light as less than 1 lux light exposure for 1 min is sufficient to trigger its translocation back to the outer segment compartment of rods.

### Phosphorylation of RGS9-1 at Ser^475^ as a possible mechanism for light-dependent RGS9-1/Gβ5L translocation in photoreceptors

It has been reported that phosphorylation of RGS proteins regulates their sub-cellular translocation [32]. RGS9-1 is expressed exclusively in photoreceptors and can be robustly phosphorylated in the dark, and light exposure decreases its phosphorylation [21]–[23]. RGS9-1 in mouse rods is phosphrylated on Ser^475^ by PKCα, and this phosphorylation significantly decreases its affinity to R9AP [22], [23]. We found that, after prolonged dark adaptation, RGS9-1 in mouse retinas isolated under 1 lux lighting conditions was phosphorylated at a level >10-fold greater than in light-adapted retinas. Furthermore, dark adaptation significantly reduced the interaction between RGS9-1/Gβ5L and R9AP. Our results agree with the earlier reports and further suggest that dark adaptation induces phosphorylation of RGS9-1, which then dissociated from R9AP. Martemyanov et al [17] reported that the DEP domain near the N-terminus of RGS9-1 is needed for RGS9-1 to associate with R9AP and without it RGS9-1 is localized primary in the inner segment compartment. Therefore, dissociated RGS9-1 should be localized to rod inner segments. Interestingly, dissociation of RGS9-1 from R9AP does not automatically lead to down-regulation of the RGS9-1 protein level. In a transgenic mutant mouse expressing a DEP-deficient RGS9-1 on a RGS9−/− background, mutant RGS9-1 expression levels appeared to be normal [17].

### Physiological implication

What is the purpose of this dark-induced re-distribution of RGS9-1/Gb5L from the outer to the inner segments? Because RGS9-1 plays a critical role in determining the duration of the photoreceponse [7], [9], [33], the most obvious purpose of this dark-induced re-distribution appears to be to increase the photoresponse duration. Dissociation of RGS9-1 from R9AP not only significantly reduces its catalytic activity on transducin; it also moves it away from transducin. Our results show that, after prolonged dark adaptation, when RGS9-1 is localized mostly in the inner segments, transducin is localized almost exclusively in the outer segments. Therefore, this newly discovered re-distribution of RGS9-1 will separate GAP activity away from transducin in order to increase the photoresponse duration for maximizing sensitivity after prolonged darkness. Our results also show that light quickly dephosphorylates RGS9-1 and resulting in re-association with R9AP. This could explain why we observed that light activates the translocation of RGS9-1 and Gβ5L to the outer segments where they work together with R9AP to terminate the photoresponse.

Though it has long been well established that prolonged dark adaptation steadily increases the sensitivity of photoreceptors [34]–[36], the mechanism underlying remains unclear. Regeneration of rhodopsin is essential for the increased sensitivity of photoreceptors after dark adaptation. But when the duration of dark adaptation is beyond a few hours, rhodopsin regeneration is insufficient to account for the further sensitivity increase [37]. Other current exist mechanism also cannot fully explain the delicate changes in sensitivity during adaptation, suggesting there may be unknown mechanism [38]–[40]. Our results suggest a possible scenario, namely that dark adaptation steadily increases phosphorylation of RGS9-1, which dissociates RGS9-1/Gb5L from R9AP and redistributes it to the inner segments. As a result, the duration of the light response could be extended to improve sensitivity at the expense of temporal resolution. It has been well documented that prolonged dark adaptation increases the susceptibility of photoreceptors to photic injury [41]–[43]. It is conceivable that heightened sensitivity to light may make photoreceptors more vulnerable to light damage and this deduction is currently under investigation. The underlying mechanism remains unclear.

### Sensitivity of RGS9-1 localization in the retina to light

Our work reveals that RGS9-1/Gb5L joins the rank of several phototransduction proteins with distinctive subcellular localizations during light/dark adaptation. Especially intriguing is its extremely low threshold of light activation required for their relocation from inner segment to the outer segment that allows photoreceptors to regulate the translocation of RGS9-1/Gb5L and transducin under different light intensities. Transducin translocation apparently requires a much higher threshold [28]. Therefore, after dark adaptation, when the retina is exposed to light between 1 to 150 lux, transducin and RGS9-1 are in the same cellular compartment. Within this light intensity range, RGS9-1 is helping transducin α hydrolyze its bound GTP to GDP to terminate the light response. Differences in the activation threshold for translocation of transducin and RGS9-1 may have important func­tional implications. After prolonged dark adaptation, RGS9-1 and transducin are in separate cellular compartments, suggesting that, after prolonged dark adaptation, the catalytic activity of RGS9-1 on transducin is reduced to increase the sensitivity to light. Due to the differences in their translocation thresholds, when the retina is exposed to a very dim light, RGS9-1 is quickly translocated to the outer segments and to join transducin. The catalytic activity of RGS9-1 on transducin is then increased to resume the speed in detecting the visual environment. Our results indicate that photoreceptor may use this dif­ference in translocation thresholds to regulate its sensitivity and speed of detection.

Translocation of RGS9-1 from the outer segments to the inner segments requires a completely dark environment, providing us with a totally new concept of dark adaptation. Usually, dark adaptation can be achieved in a dark room with dim light or even a dim red light. Especially, in most circumstances, retinas are isolated and processed under dim red light. Our results indicate that, after prolonged complete dark adaptation, very dim light has already induced significant changes in rod photoreceptors, setting a new paradigm for studying more subtle changes in photoreceptors. It will be interesting to re-examine the localization of other phototransduction proteins by adopting this more stringent dark adaptation condition. Similarly it will be informative to examine whether such lighting conditions affect the distribution of RGS9-1 in cones. Such studies are currently in progress.

The major finding of our study lies in the unappreciated and distinct localization of RGS9-1/Gβ5L and transducin in separate cellular compartments of rod photoreceptors in completely dark-adapted mouse retina. This suggests the presence of a novel mechanism to regulate the rate-limiting step of recovery of rod phototransduction, allowing rod photoreceptors to trade temporal resolution for sensitivity and may thus be important for scotopic vision.
